# Supplementary material for: Dual enzyme-powered chemotactic cross β amyloid based functional nanomotors
Source: Nat Commun. 2023 Sep 22;14:5903. doi: 10.1038/s41467-023-41301-x (PMC10516904; doi:10.1038/s41467-023-41301-x)
Supplement: Supplementary file 1 — Supplementary Information [file 41467_2023_41301_MOESM1_ESM.pdf]

Supplementary Information

Dual Enzyme-powered Chemotactic Cross β Amyloid based Functional Nanomotors

Chandranath Ghosh[1,5], Souvik Ghosh[1,5], Ayan Chatterjee[1], Palash Bera[2], Dileep Mampallil[3], Pushpita Ghosh[4], Dibyendu Das[1]*

[1]Department of Chemical Sciences and Centre for Advanced Functional Materials, Indian Institute of Science Education and Research (IISER) Kolkata, Mohanpur 741246, India.

[2]Tata Institute of Fundamental Research (TIFR) Hyderabad, Telangana 500046, India.

[3]Department of Physics, Indian Institute of Science Education and Research (IISER) Tirupati, Mangalam, Andhra Pradesh, 517507, India.

[4]School of Chemistry, Indian Institute of Science Education and Research (IISER) Thiruvananthapuram, Kerala, 695551, India.

[5]These authors contributed equally: Chandranath Ghosh, Souvik Ghosh.

E-mail: dasd@iiserkol.ac.in

Motion study and experimental procedure

Motion analysis

Motion analysis of amyloid-based nanomotors was done by using the standard protocol.[1] An Olympus IX81 and IX83 inverted microscope equipped with a 60x (water objective) and 100x (oil objective) was used to observe the motion of amyloid-based nanomotors. The movies were recorded using Cool SNAP MYO CCD camera and Hamamatsu Orca Flash 4.0 LT+ in differential interference contrast (DIC) mode (frame interval 100 ms). Aliquots of amyloid-based nanomotors were mixed thoroughly with varying concentrations of fuel urea (0, 25, 50, 100 mM) and subsequently casted onto cavity slides. Slides were sealed with coverslips to prevent evaporation and air bubble formation to avoid convective flows. Further, amyloid-based nanomotors were manually traced using ImageJ. A python code was developed to get mean-squared displacements from respective trajectories of the nanomotors which are provided in Figs. 3e and Supplementary Fig. 6 and tracked from Supplementary Movies 1 to 4. Further to obtain insights into motion types, MSD vs time interval was plotted in Origin graphing software and fitted using the following equations:

$\mathbf{1.}\,\boldsymbol{MSD} = <|\boldsymbol{x\_i}\,(\boldsymbol{t}+\boldsymbol{\Delta t}) - \boldsymbol{x\_i}\,(\boldsymbol{t})|\mathbf{2}>$, (value of i is taken 2 for 2D analysis).
$\mathbf{2.}\,\boldsymbol{MSD} = \mathbf{4}\boldsymbol{D\Delta t} + (\boldsymbol{v\Delta t})\mathbf{2}$ [parabolic fitting that represents a non-Brownian type active motion, Fig. 3e, red and green line]
$\mathbf{3.}\ \boldsymbol{MSD} = \mathbf{4}\boldsymbol{D\Delta t}$ [linear fit represents Brownian motion, Fig. 3e, black line in manuscript]
D represents the effective diffusion coefficient, Δt represents the time interval.

To avoid error, averages of MSD values were taken for multiple nanomotors (20 particles were tracked) as MSD signatures tend to vary quite significantly in the case of individual nanomotors. As a result, errors were incorporated into the corresponding MSD signatures along with extracted velocities.

For understanding the spatiotemporal dynamics of amylobots, the MSD values were fitted in the eq. $MSD = 4D\Delta t^{\alpha}$, where Δt is the time interval, D is the diffusion constant and α is the MSD exponent. Depending upon MSD exponents, we characterized the type of diffusion i.e. how fast or slow the particles are diffusing in the space.[2,3] For $\alpha = 1$ indicates the Brownian diffusion, $\alpha < 1$ implies the sub-diffusion, and $\alpha > 1$ specifies the superdiffusion. Supplementary Fig. 11 demonstrates the fitting of experimental and simulated MSD plots as a function of time intervals respectively. Here scatter points and dotted lines are original (experimental or simulated) and fitted data respectively. From these two plots, it becomes clear that in the absence of urea (motility force), the nanotubes are showing Brownian diffusion ($\alpha \sim 1$). However, for higher values of urea concentration (motility force), the amylobots are showing superdiffusive motion ($\alpha > 1$) suggesting urea concentration (motility) driven fast dispersal.

Experimental design for investigating chemotaxis

1% agarose gel (1g/100mL) was prepared by mixing agarose with water followed by heating onto a hot plate to boiling temperature for five minutes. Next, the hot agarose solution was poured onto a round petriplate. From that, a rectangular portion (2 cm ×1.5 cm ×1 cm) was scooped out. A pyrogallol reservoir was prepared via adding pyrogallol in one side of the agarose gel by making a hole with a glass capillary tube (marked as 'P' in Figure 4a). The scooped portion was filled with buffer (10 mM HEPES, pH 7.0) to create a pyrogallol gradient where H_2O_2 was distributed throughout the buffer (final concentration = 20 mM). Amyloid-based nanomotors loaded with cytochrome c (CytC) and urease (Ac-KL=100 µM, [urease] = 20 µM, [CytC] = 20 µM) were added at point 2 (Fig. 4a, Supplementary Fig. 13). Aliquots were taken out from point 1 and 3 at different time intervals from (Fig. 4a, Supplementary Fig. 13) and estimated the urease concentration bound to nanotubes by using the Urease activity assay following Berthelot method.[4,5] Controls were done by following a similar protocol. Notably, altering the location of the substrate reservoir did not impact the chemotactic motility which was always towards the chemical cue (pyrogallol).

For the other setup, a pyrogallol (substrate of cytochrome C) reservoir was prepared via adsorbing pyrogallol in agarose gel media. Briefly, 1% agarose gel (1g/100mL) was prepared, and 1/3rd volume of the petriplate was filled and kept for cooling. Aliquot of pyrogallol (1M stock) was added dropwise to adsorb on the gel matrix (final concentration 10 mM). The other 2/3rd volume was filled with buffer (10 mM HEPES, pH 7.0) to create a pyrogallol gradient where H_2O_2 was distributed throughout the buffer (final concentration = 20 mM). Amyloid-based nanomotors loaded with urease and CytC (Ac-KL=100 µM) were added at position 1 (Supplementary Fig. 20). Aliquots were taken out at different time intervals from a point close to the gel/buffer interface (at point 3 in Supplementary Fig. 20) and added to a microvolume quartz cell (10 mm) to monitor the oxidation at 420 nm (ε_{420} nm is 2640 $M^{-1}cm^{-1}$ in buffer).[6] Controls were done following a similar protocol.

Control experiment with orthogonal enzyme

To rule out any possible drift on nanotubes that could have happened by the chemical cue released from the agarose gel we have performed control experiments with the orthogonal enzyme. As an orthogonal enzyme, we have taken Glucose Oxidase (GOx) and monitored the population of the nanomotors (nanotubes + urease) in a similar experimental setup used to show chemotaxis (Supplementary Fig. 19a). Interestingly, no directional bias was observed (Supplementary Fig. 19b) as seen in the case of urease-CytC-loaded nanotubes (Fig. 4b).

Measurement of nanomotor population during chemotaxis

To monitor chemotaxis, two separate setups were used. For the first setup, the population of urease-bound nanotube was tracked using the Berthelot method[4,5] (Fig. 4a, Supplementary Fig. 13). In the

presence of urease, urea will be hydrolyzed and produce ammonia (NH_3) and CO_2, and the ammonia generated was estimated following the below-mentioned equations. 10 μL of aliquots from positions 1 and 3 were pipetted out (Fig. 4a, Supplementary Fig. 13) at different times. To the pipetted-out reaction mixture, a saturated amount of urea with salicylate and hypochlorite was added along with nitroprusside, and the absorbance of the greenish-coloured indophenol formed was monitored at 660 nm. From a standard plot at 660 nm using a saturated concentration of urea, the amount of the urease present was estimated. The absorbance was measured using Cary 3500 (G9864A) UV-Vis spectrophotometer and the experiments were performed in triplicates.

$$\text{Urea} + H_2O \longrightarrow 2NH_3 + CO_2$$

$$NH_4^+ + \text{Salicylate} + \text{NaOCl} \xrightarrow[OH^-]{\text{Nitroprusside}} \text{Indophenol} + \text{NaCl}$$

For the second setup, in a round petriplate as mentioned above (Supplementary Fig. 20), native urease mixed with RITC-tagged urease (9:1 molar ratio) was used for loading onto the amyloid nanomotors containing CytC. Aliquots (100 μL) were taken out from the three different positions using a multi-pipette and fluorescent intensities were measured (BioTek, SYNERGY H1, Supplementary Fig. 21).

Experimental design for the microscopic observation of nanomotor population during chemotaxis

To check the directional motility of the nanomotors towards a chemical cue, a new type of setup was used. Briefly, 1.5 cm long glass capillaries were used, and the capillaries were sealed at one side and then filled with different concentrations of the chemical cue pyrogallol (0 mM, 50 mM, 100 mM) and the open side of the capillaries were placed at 35 mm glass bottom petriplate (Fig. 4c, Supplementary Fig. 24). The petriplate was filled with buffer containing H_2O_2 and urea. Dual enzyme-loaded nanomotors were added to the buffer medium. The field of view of the microscope was fixed at the open end of the capillaries and then monitored the population of the nanomotors with time. After 15 min, the population of the nanomotors was observed to be significantly higher for the highest concentration of pyrogallol due to the chemotactic movement of the nanomotors.
To further visualize the chemotaxis, a similar experiment was performed under confocal microscopy when CytC and urease were loaded on the nanomotors along with RITC-tagged urease (9:1 molar ratio). Localization of the nanotubes was found to be significantly increased at the capillary opening with the increase of the pyrogallol concentration (Supplementary Fig. 25). In the absence of the pyrogallol, the localization of the nanotubes was significantly diminished.

$$\vec{v}_i = \frac{1}{\eta L_i}\vec{F} = \frac{1}{\eta L_i}(\vec{F}_{rf} + \vec{F}_{mf} + p\vec{G})......(1)$$

$$\omega_i = \frac{12}{\eta L_i^3}\tau......(2)$$

Activity measurement of CytC loaded nanomotors in toluene medium:

The peroxidase activity of CytC-bound amyloid-based nanomotors was monitored both in the HEPES buffer (pH 7.0) as well as in the toluene system by using Cary 3500 (G9864A) UV-Vis spectrophotometer. Amyloid-based nanomotors were prepared using the protocol mentioned in the above section.[7,8] Afterwards, 25 µL of the CytC-urease-amyloid nanohybrids was added to a round bottom (RB) flask followed by the addition of toluene (25 mL). After stirring for ca. 1 min, 83.3 µL of pyrogallol (3M stock in acetone) was added.[7] The final concentration of the pyrogallol was 10 mM. Further, varying concentrations of urea (0, 25, 50, and 100 mM) were added to the RB flask to check the effect of motility on catalytic performance. Finally, to initiate the peroxidase reaction, the required amount of H_2O_2 solution (9.8 M stock) was added to the stirred solution of organic solvent (the final concentration of H_2O_2 was 60 mM). Aliquots were taken from the upper half of the reaction solvent to monitor the absorbance of the product. The increase of absorbance at 420 nm (production of purpurogallin, ε_{420} nm is 4400 $M^{-1}cm^{-1}$ in toluene) was measured at definite intervals. It is important to note here that native CytC shows 3 orders of magnitude lower activity in a similar setup with toluene in the absence of the nanomotors. Notably, separate controls showed no role of urea in augmenting CytC activity in pH-controlled conditions.

Computational Study

a) Particle-based model of amyloid-based nanomotors as self-propelling nanotubes

In order to get a mechanistic understanding of the movements of amyloid-based nanomotors nanotubes as found in the experiments, here we propose an individual-based/particle-based model describing each amyloid nanotube as a cylindrical particle of fixed diameter 50 nm and varying lengths between (200-600) nm. The length of the nanotubes chosen in the simulations corroborates to the experimentally observed values (length = 180-560 nm), however, for computational ease, we kept the diameter slightly larger than the experimental values (diameter = 32 $\pm \mathbf{2}$ nm). Supplementary Fig. 8 schematically illustrates the length L and the diameter $\boldsymbol{d_0}$ of a nanotube. The equations of motion of individual nanotubes are described by equations (1) and (2), which follow an over-damped dynamics, where viscosity dominates over inertia and linear velocity and angular velocity proportional to force and torque respectively.

Where $\vec{v}$, ω, η, τ, and L are the linear velocity, angular velocity, friction co-efficient, torque, and length of the nanotube respectively and i is the index of the corresponding nanotube. Here, we have considered a short-range repulsive interaction among the nanotubes if they interact as amyloid-based nanomotors are found to repel each other and have neglected any kind of long-range interaction. Each nanotube experiences a total force which is summation of repulsive force $\vec{F}_{rf}$, motility force $\vec{F}_{mf}$ and a random noise contribution $p\vec{G}$ from the surrounding. Here G describes the Gaussian random noise with a zero mean and standard deviation = 1.0 and p denotes the strength of the noise. For simulation purpose, we have chosen a length-dependent coefficient as $p = A\sqrt{L}$, where A is a constant and L is the length of the nanotube. Mechanical interaction between two nanotubes is assumed in accordance with the Hertzian theory of elastic contact which efficiently describes the repulsive interaction between two elongated rod-like particles as used in previous studies[9], as is given by $F_{rf} = E\, d_o^{1/2} h^{3/2}$, where E , d_0 and h represents Elastic coefficient, diameter, and overlap between two interacting nanotubes respectively, as depicted in Supplementary Fig. 9, where $h = d_o - r_0$ and r_0 is the closest distance of approach between two nanotubes.

In our simulations, we begin with inoculating a mixture of 200 nanotubes, with length ranging in between $(0.2 - 0.6)\mu m$, at the center of a two-dimensional simulation box of size $(200 \times 200)\mu m^2$, randomly, in a circular inoculation. The origin of the motility force of each nanotube amyloid based nanomotors underlies an enzymatic reaction between the enzyme urease with the urea present in the system. The self-propelling motion increases with the increase of available urea concentration in the mixture. To mimic the experimentally observed variation of motility as a function of urea concentration, in simulations, we have varied the motility force $|\vec{F}_{mf}| = f_u$ in analogous to experiment. The spatiotemporal dynamics of the nanotubes are obtained by integrating the equations of motion, equation (1) and (2) by using the Euler scheme with a time step $dt = 0.001$ and dumped the data in each 100 steps (data dumping frequency).

b) A unified model of amyloid based nanomotors nanotubes to capture the chemotactic movement

In order to simulate the chemotactic motion of the amyloid based nanomotors nanotubes, we performed simulations in a rectangular box of size $400 \times 200\ \mu m^2$. $1/3^{rd}$ Portion of the box is filled with pyrogallol and a pyrogallol reservoir at three sides is maintained, where we have placed pyrogallol initially. A circular inoculation of 200 nanotubular particles of varying lengths $(0.2 - 0.6)\mu m$ is placed at a position $x = L_x \times \frac{2}{3}$, $y = L_y \times \frac{1}{2}$. The substrate pyrogallol can diffuse following a diffusion equation as given in equation (3).

$$\frac{\partial C}{\partial t} = D\left[\frac{\partial^2 C}{\partial x^2} + \frac{\partial^2 C}{\partial y^2}\right] \text{.........(3)}$$

Where C and D are the concentration and Diffusion constant of pyrogallol respectively. The spatio-temporal evolution of individual amyloid based nanomotors nanotubular particle follows a generalized/unified equation of motion as below (equation (4) and equation (5)):

$$\vec{v}_i = \frac{1}{\eta L_i}\left[\vec{F}_{rf} + \alpha\vec{f}_u + (1-\alpha)\left(f_u f_c \chi_1 \vec{\nabla} C + \chi_2 f_c \vec{\nabla} C\right) + p\vec{G}\right] \text{............(4)}$$

$$\omega_i = \frac{12}{\eta L_i^3}\tau \quad \text{......(5)}$$

Where χ_1 and χ_2 are the chemotaxis coefficient that maintains the dimension of the equation of motion. f_u,f_c, and ∇C are the motility forces due to urease and CytC and gradient of pyrogallol concentration respectively with condition $f_u \gg f_c$. For simulation purpose, we have chosen $\chi_1 = 1.0$ and $\chi_2 = 1.0$. There are three different possibilities, (i) $\alpha = 0 \Longrightarrow$ both contribution of urease and CytC (chemotaxis), (ii) $\alpha = 1 \Longrightarrow$ only urease contribution (different urea concentration), (iii) $f_u = 0$ and $\alpha = 0 \Longrightarrow$ (CytC contribution). Here α behaves like a switch with on and off state provides different types of scenarios.

In order to examine angular diffusion, we computed the rotational mean squared displacement (MSD) for both with and without the presence of CytC, following the simulation setup described in Figs. 4(d-g). The rotational MSD is defined as:

$$\Delta^2\, \theta(\Delta t) = |\theta(t + \Delta t) - \theta(t)|^2 \quad \text{......(6)}$$

where θ represents the orientation of the nanotubes and Δt denotes the time interval. Supplementary Fig. 32 shows the rotational MSD of the nanotubes over time, in the presence and absence of CytC. From the figure, it is evident that the rotational MSD is greater in the absence of CytC compared to its presence, indicating the unidirectional movement of the nanotubes when CytC is present.

c) Simulation Parameters

Parameters	Symbol	Simulations
Length of nanotubes	L	$(0.2-0.6)\mu m$
Diameter of nanotubes	d_0	$0.05\ \mu m$

Elastic modulus of nanotubes	$\boldsymbol{E}$	$\mathbf{1.5 \times 10^9}\ \boldsymbol{Pa}$ Ref [10]
Friction co-efficient of nanotubes	$\boldsymbol{\gamma}$	$\mathbf{7.5 \times 10^5}\ \boldsymbol{Pa.s}$
Motility Force (urease)	$\boldsymbol{f_u}$	$\mathbf{(0-24) \times 10^5}\ \boldsymbol{Pa.\mu m^2}$
Concentration (pyrogallol)	$\boldsymbol{C}$	$\mathbf{50}\boldsymbol{fg/\mu m^3}$
Diffusion constant	$\boldsymbol{D}$	$\mathbf{800}\boldsymbol{\mu m^2/s}$
Motility force (CytC)	$\boldsymbol{f_c}$	$\mathbf{10}\boldsymbol{Pa.\mu m^2}$
Strength of random noise	$\boldsymbol{A}$	$\mathbf{2.7 \times 10^7}$

Experimental techniques

Transmission Electron Microscopy Imaging (TEM)

For structural characterization purpose, peptide assemblies of mature and sonicated were adsorbed for 1 min on the TEM grid followed by wicking off excess peptide solution by filter paper. Next, 2% (w/v) uranyl acetate was added and incubated for 2 minutes. Samples were then placed in desiccator under vacuum for complete drying. TEM studies were orchestrated with a JEOL JEM 2100 with a tungsten filament with accelerating voltage of 200 kV**.** For the characterization of the enzyme distribution on the nanotube surface, uranyl acetate was not added.

Scanning Electron Microscopy Imaging (SEM)

Preparation of SEM samples followed similar protocol as followed for TEM sample preparation. Briefly, samples were casted on silicon wafer and dried completely before imaging. SEM images were recorded on a Carl Zeiss SUPRA 55VP instrument.

Atomic Force Microscopy (AFM)

Peptide assemblies were diluted to a final concentration of 150 μM. 20 μL of aqueous peptide solutions was casted on a silicon wafer, previously cleaned by sonication in methanol for 20 min. This was followed by adsorption on a silicon wafer for 1 min. Extra peptide solution was wicked off. Finally, the samples were vacuum-dried and scanned using Oxford MFP-3D bio-AFM. Similar procedures were maintained for sonicated samples as well.

Circular Dichroism (CD)

The CD measurements were done in a JASCO J-810 circular dichroism spectrometer at RT (maintained via Peltier temperature controller). Before running the sample, milli-Q water was used for baseline correction. 500 μL of the peptide samples were taken into a quartz cuvette (2 mm path length). To record each spectrum, scanning wavelengths from 600 nm to 190 nm was used at a scanning rate of 50 nm/min. Three successive wavelength scans were performed and averaged at the end.

Fourier-Transform Infrared Spectroscopy

IR spectra were recorded using Bruker (model no: Alpha) in ATR mode (Platinum ATR) at RT by averaging 256 scans with 4 cm^{-1} resolutions. In this context, sample aliquots were dried properly as a thin film. Background spectra were subtracted from the spectrum.

Confocal microscopy

RITC tagged urease and FITC tagged CytC were co-localized onto nanotubular surface. In this context, these fluorophores tagged enzymes were incubated with 1 mM of Ac-KL and further casted on glass slide and sealed using coverslip. Similar procedure was also done for only RITC labelled urease localization. Apart from enzyme binding visualization, Nile red binding study was also performed to probe the nanotubular surface. All the confocal images were captured by CLSM 710 (ZEISS Axio observer 2.0), with 12-bit depth with a 100X, Plano Apochromat objective (N.A.1.4), excited by 488 nm, 561 nm laser line.

For distinguishing the migration behaviour of the active (urease-CytC-loaded) and the passive (only urease-loaded) systems, nanotubes were labelled with different fluorophores. FITC and RITC were used for labelling the active and passive nanotubes respectively. All the fluorescent intensity measurement study was performed using the Olympus Laser Scanning Confocal System Model FV3000 with a 10X objective [part of the Atomic Force Microscope with Rheological Measurement and Confocal Imaging Unit Facility, supported by Swarnajayanti (SB/SJF/2020-21/08)].

Supplementary Figures

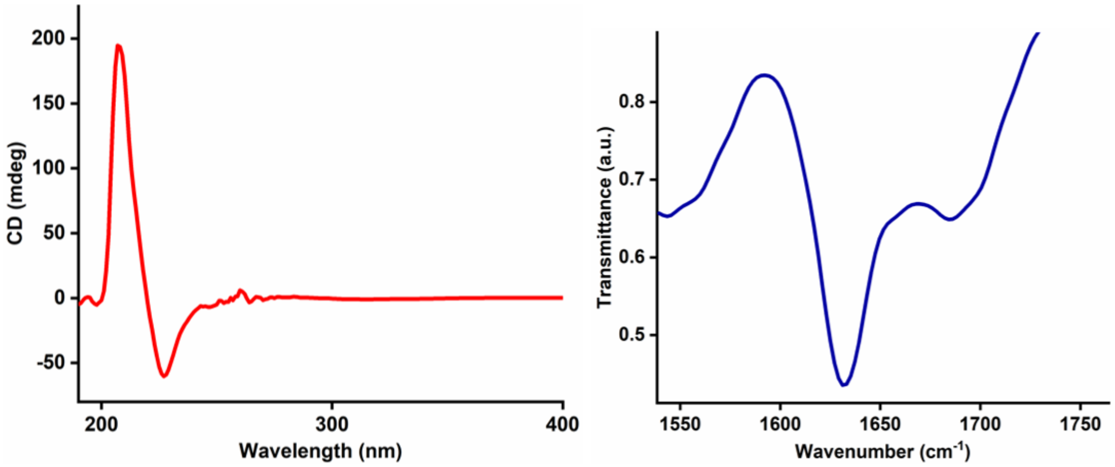

Supplementary Figure 1. CD spectrum (left) and FTIR spectrum (right) of amyloid Ac-KL nanotubes.

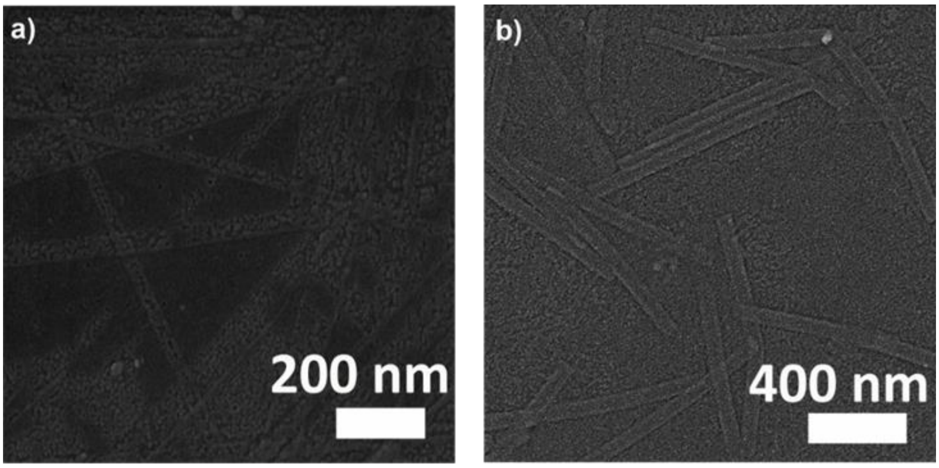

Supplementary Figure 2. SEM micrographs of Ac-KL nanotubes: (a) non-sonicated; (b) sonicated.

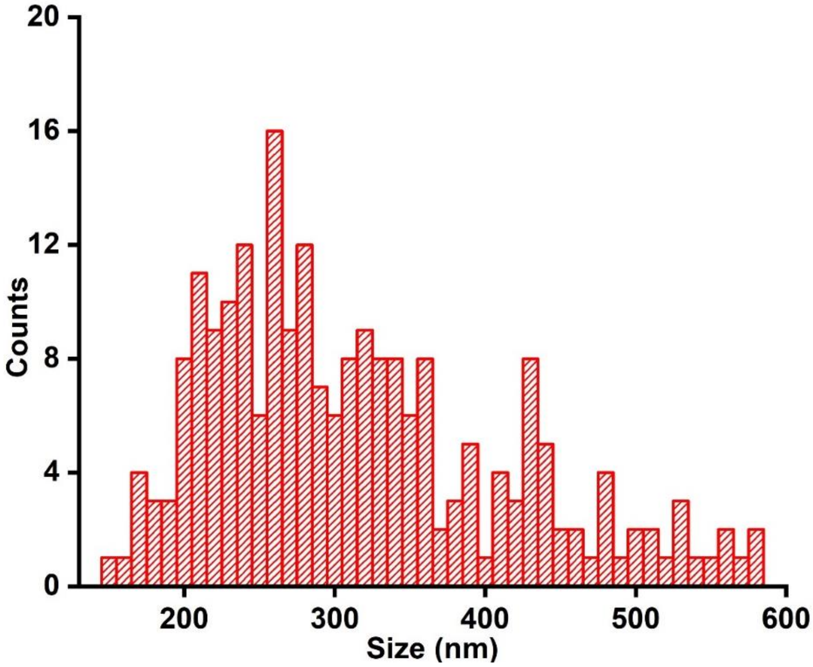

Supplementary Figure 3. Size distribution profile of sonicated Ac-KL nanotubes.

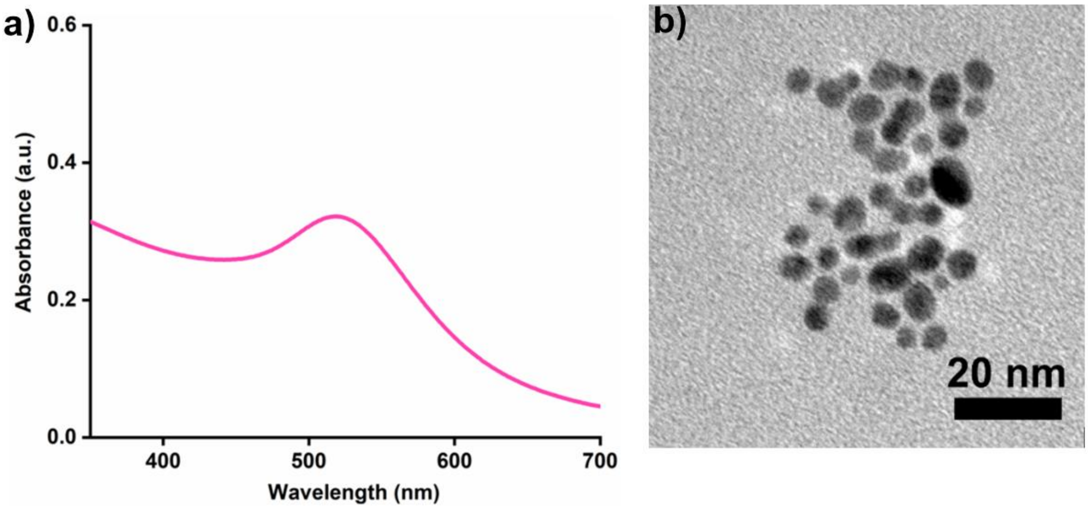

Supplementary Figure 4. (a) UV-vis spectra representing localized surface plasmon resonance (SPR) transition and (b) TEM micrographs of negatively charged GNps.

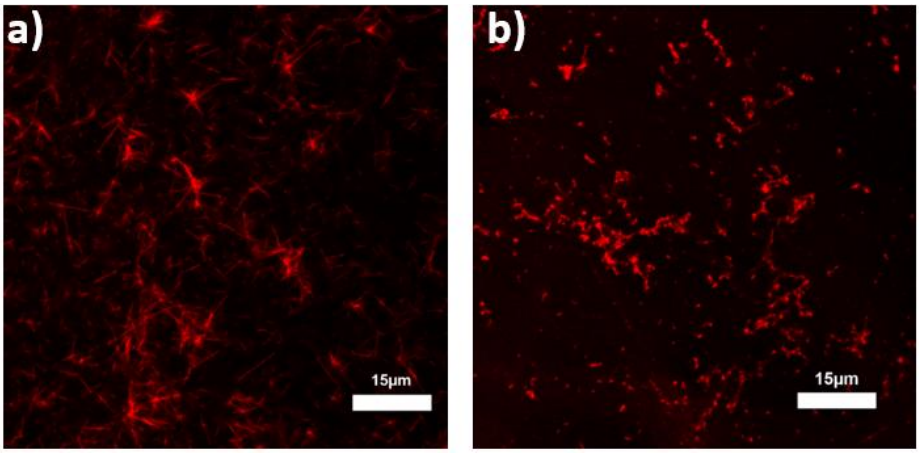

Supplementary Figure 5. Confocal micrographs of Nile red bound Ac-KL nanotubes, (a) non-sonicated; (b) sonicated.

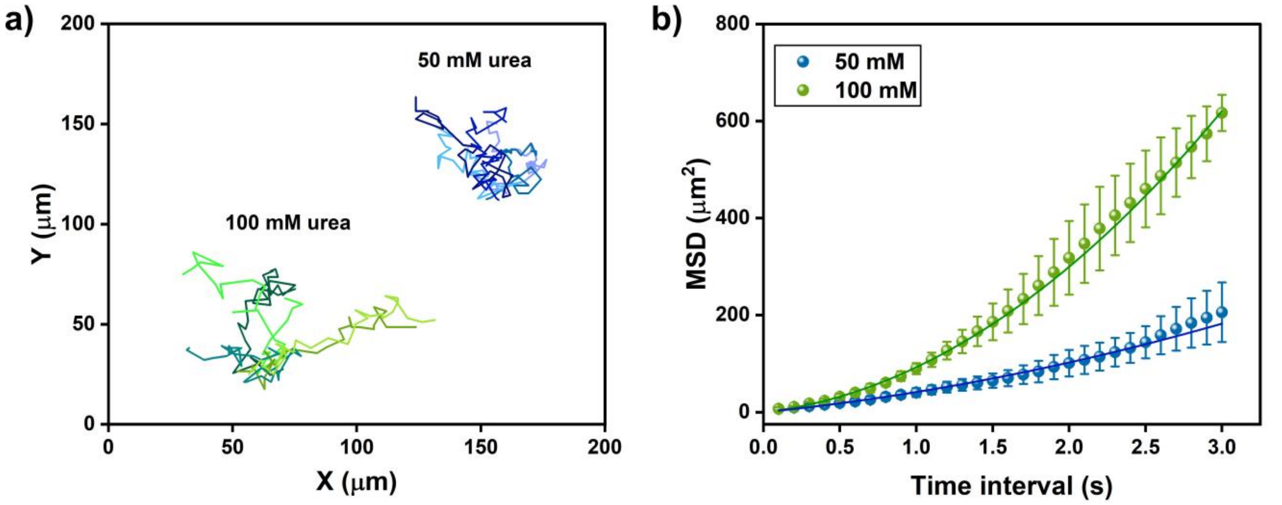

Supplementary Figure 6. (a) Tracking trajectories and (b) MSD vs time interval of the nanomotors with varying urea concentration (tracking analysis of 5 particles in the same ROI as shown in Figs. 3d,e).

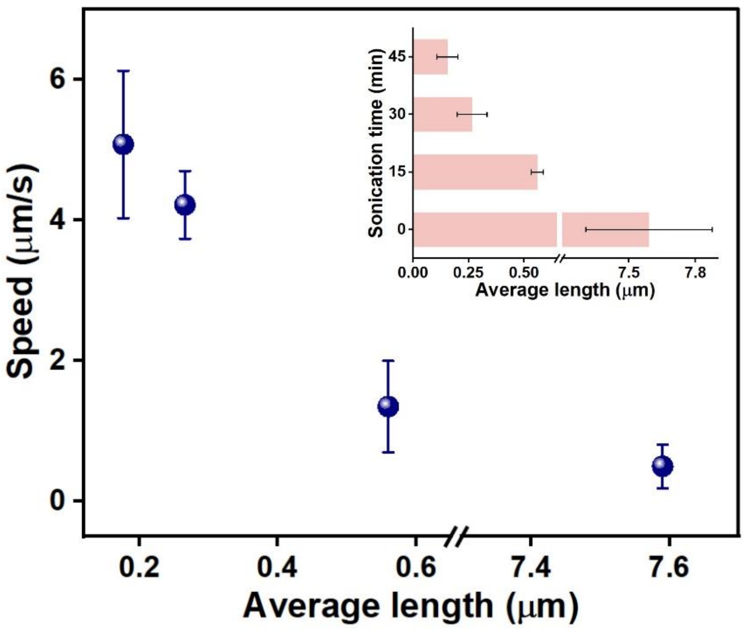

Supplementary Figure 7. Length dependence of the speed of the nanotubes (inset shows the average length under different sonication time). The error bars were calculated from three separate experiments.

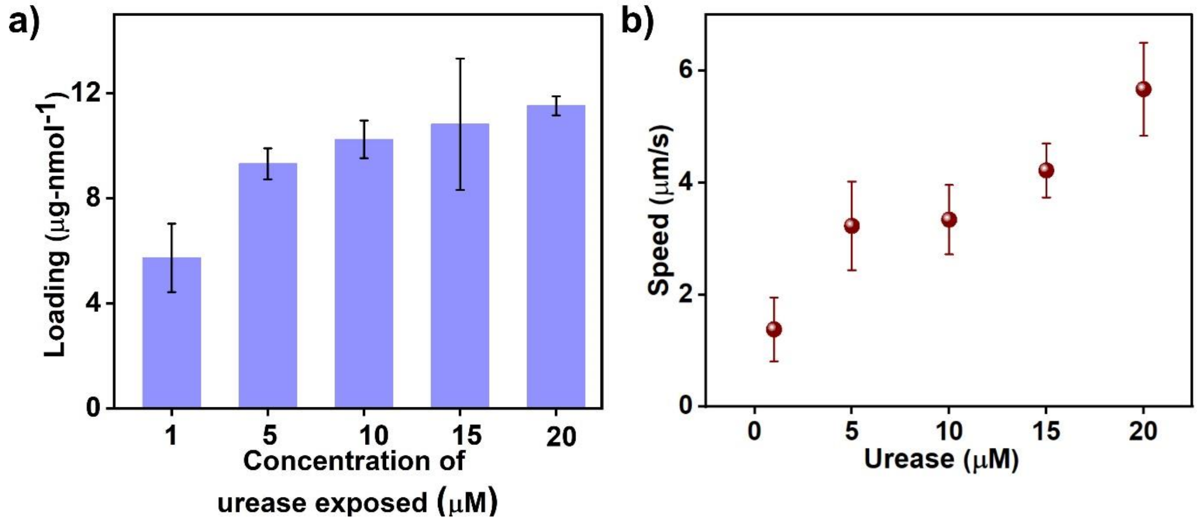

Supplementary Figure 8. (a) The loading of urease on nanotubes with varying concentration of exposed urease. (b) Loading dependence of the velocity of the nanotubes. The error bars were calculated from two separate experiments.

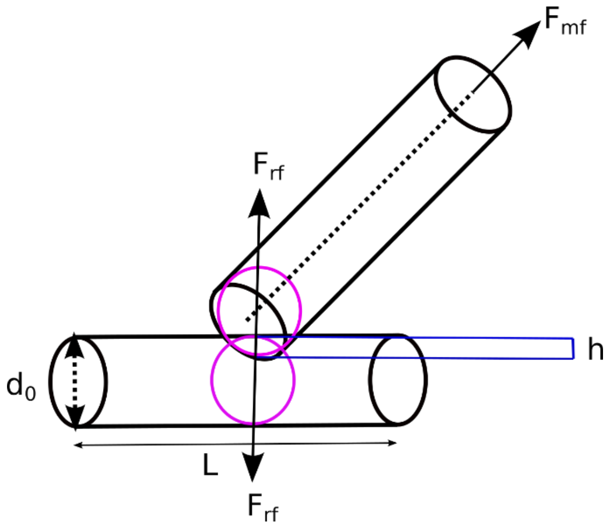

Supplementary Figure 9. Schematic of nanotube with length L and diameter d_0. F_{rf}, F_{mf} and h are the steric repulsive force, motility force and overlap between two nanotubes respectively.

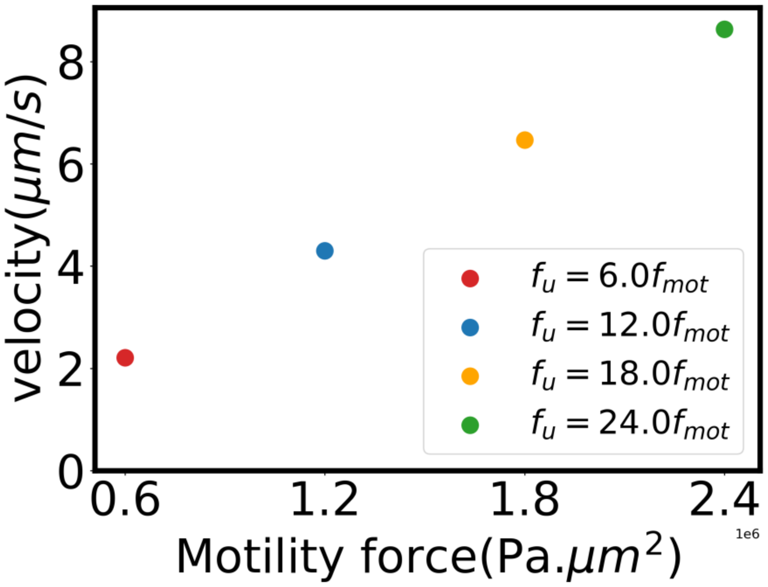

Supplementary Figure 10. Velocity as a function of motility force derived directly from the simulation results.

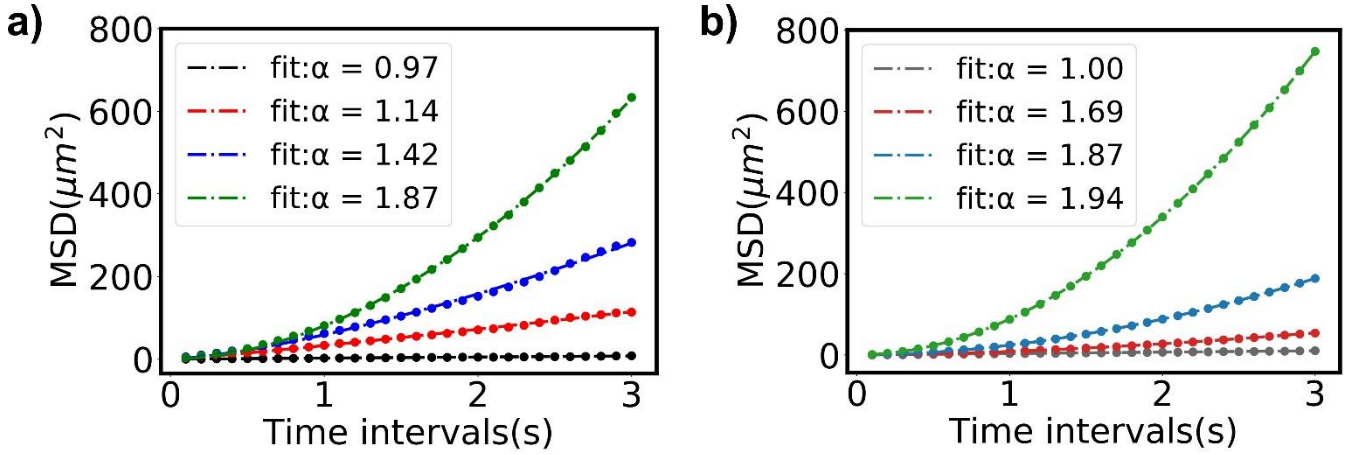

Supplementary Figure 11. Demonstration of the superdiffusive motion of the nanotubes from the MSD analysis of (a) experimental and (b) simulated systems. The dotted lines represents the fit of the Δt values by the eq. MSD = 4DΔt$^{\alpha}$, where Δt is the time interval, D is the diffusion constant and α is the MSD exponent.

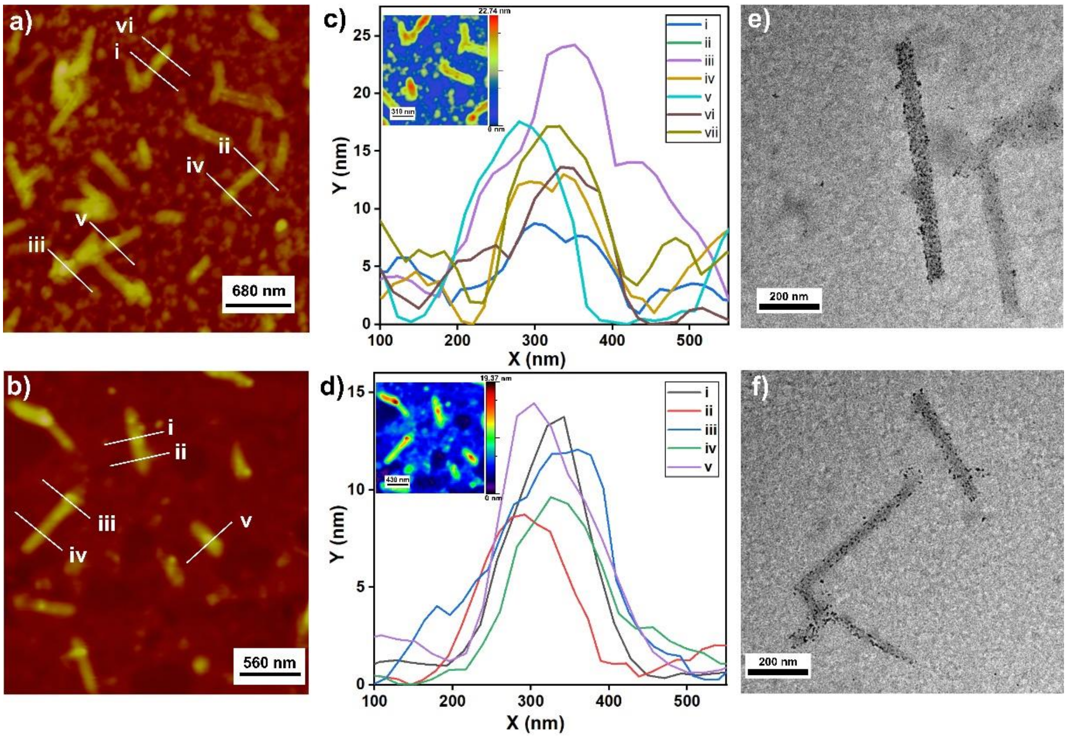

Supplementary Figure 12. (a,b) AFM micrograph of urease and CytC bound and only CytC bound sonicated nanotubes and (c,d) corresponding line analyses (insets show color-coded AFM images for variable heights) and (e,f) TEM images.[11]

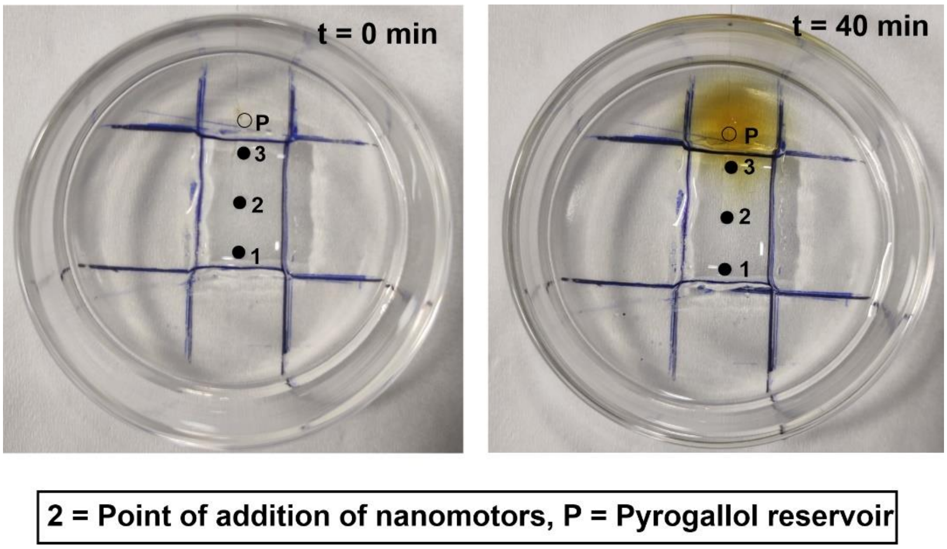

Supplementary Figure 13. Petri plate images of the set up for the estimation of ureases-bound nanotube at different time point (0, 40 min).

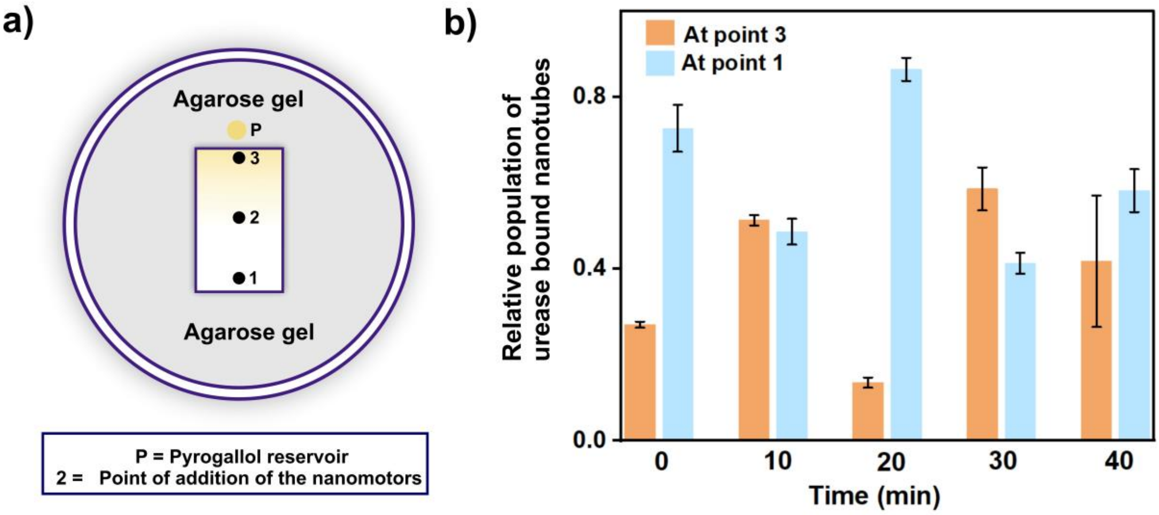

Supplementary Figure 14. The control experiment without CytC. (a) Schematic representation of the experimental set up. (b) Time dependent relative population of the enzyme loaded nanomotors. The error bars were calculated from three separate experiments.

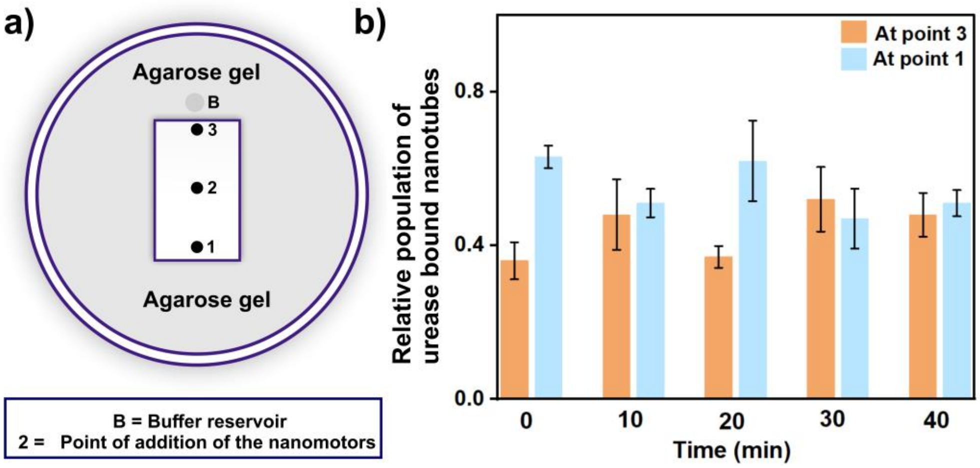

Supplementary Figure 15. The control experiment without pyrogallol. (a) Schematic representation of the experimental set up. (b) Time dependent relative population of the enzyme loaded nanomotors. The error bars were calculated from three separate experiments.

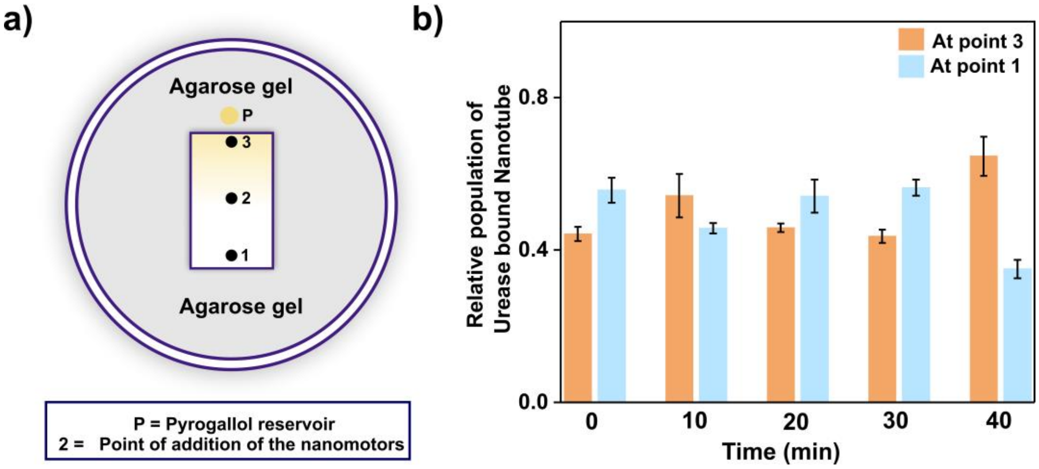

Supplementary Figure 16. The control experiment for free CytC with urease loaded nanotube. (a) Schematic representation of the experimental set up. (b) Time dependent relative population of the enzyme loaded nanomotors. The error bars were calculated from three separate experiments.

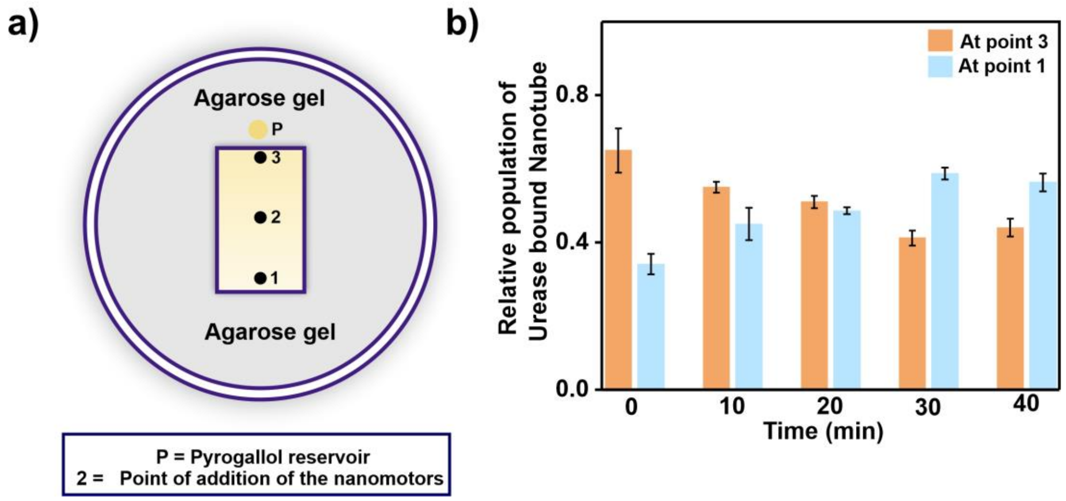

Supplementary Figure 17. The control experiment with the gel equilibrated with pyrogallol. (a) Schematic representation of the experimental set up, (b) Time dependent relative population of the enzyme loaded nanomotors. The error bars were calculated from three separate experiments.

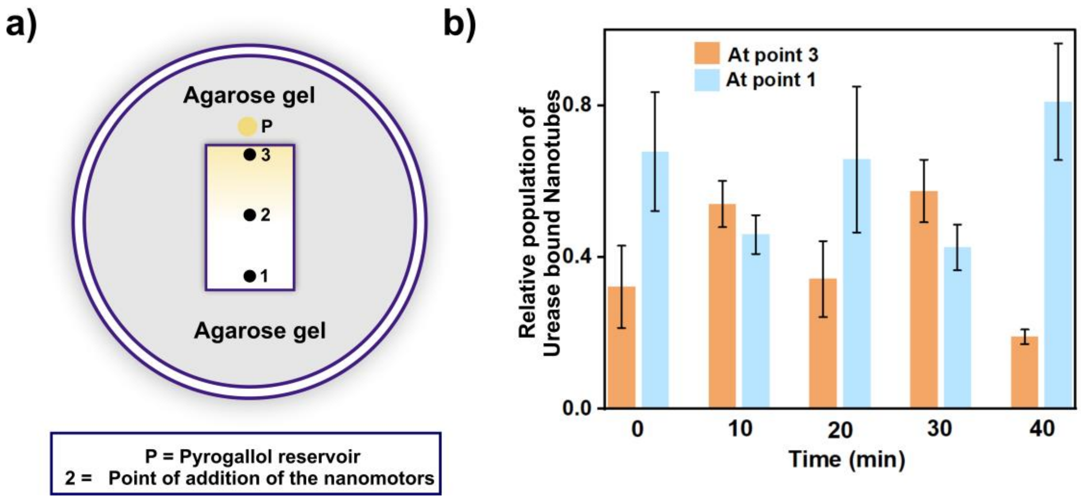

Supplementary Figure 18. The control experiment for free CytC added in the gel with the pyrogallol. (a) Schematic representation of the experimental set up, (b) Time dependent relative population of the enzyme loaded nanomotors. The error bars were calculated from three separate experiments.

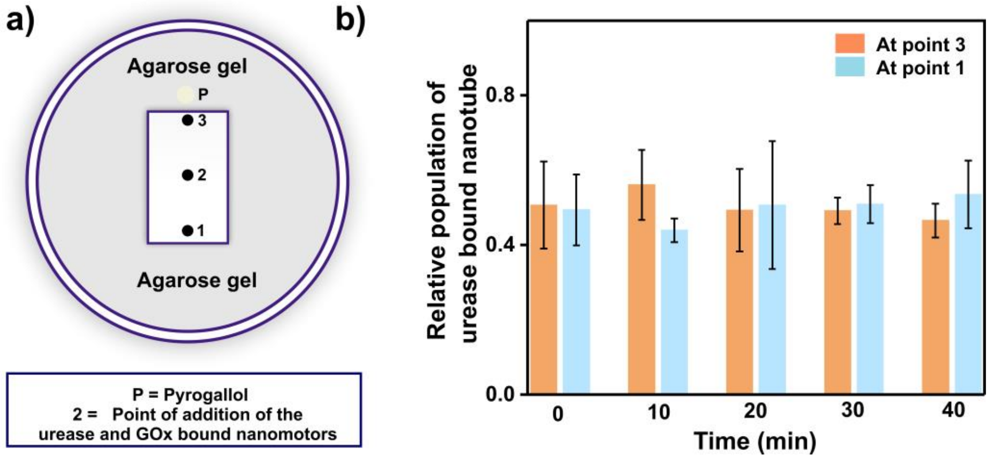

Supplementary Figure 19. The control experiment by using an orthogonal enzyme (GOx) to rule out any external drift. (a) Schematic representation of the experimental setup. (b) The time-dependent relative population of urease and GOx bound nanomotors at points 1 and 3. The error bars were calculated from three separate experiments.

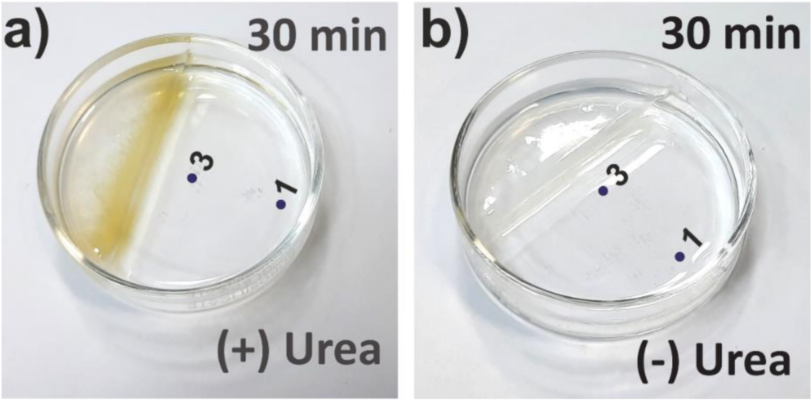

Supplementary Figure 20. Petri plate images of the urease-CytC system at different time point (after 30 min) (a) in presence and (b) in absence of urea.

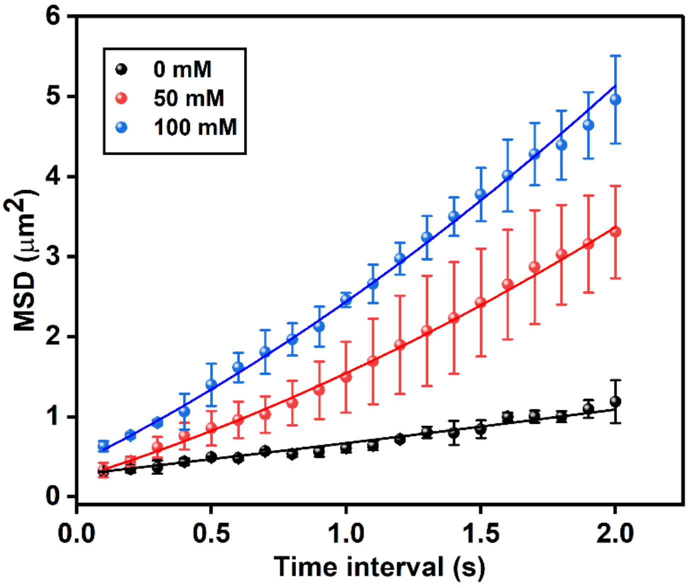

Supplementary Figure 21. MSD vs time interval plots of CytC bound sonicated Ac-KL nanotubes in presence of different pyrogallol concentrations.

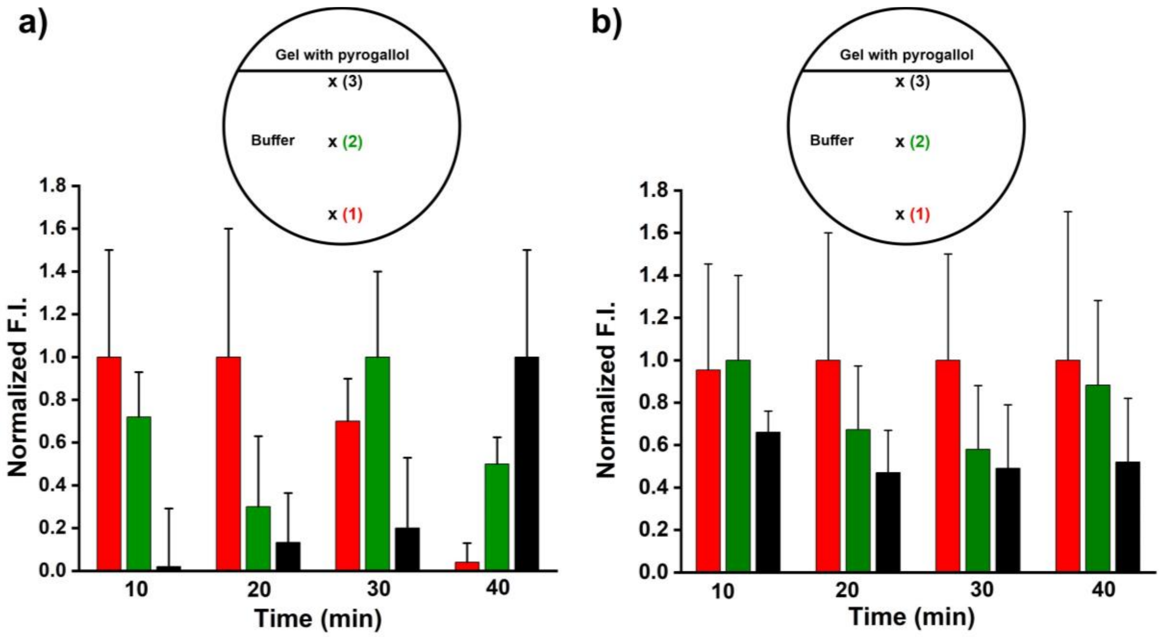

Supplementary Figure 22. Time course shift in normalized fluorescence intensity of system at different points in petri plate (red: 3; green: 2; black: 1, at point 1 the nanomotors were added) (a) in presence of urea and (b) in absence of urea. The error bars were calculated from three separate experiments.

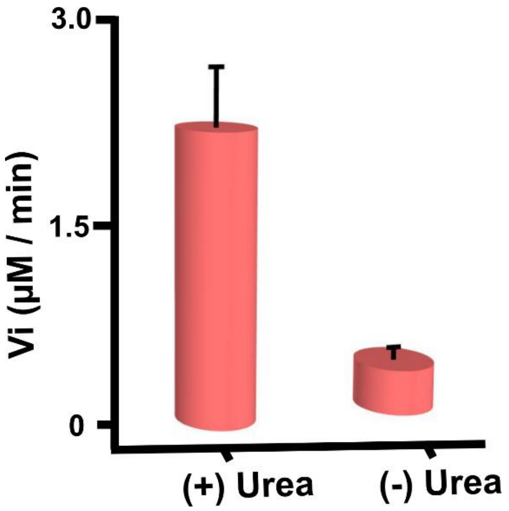

Supplementary Figure 23. Bar diagram corresponding to oxidation rates of pyrogallol in presence and absence of urea at point ‘3’. The error bars were calculated from three separate experiments.

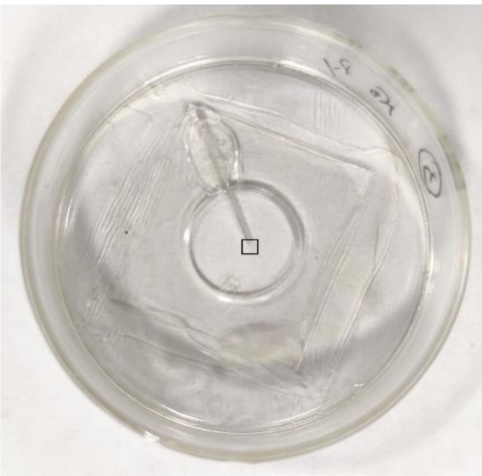

Supplementary Figure 24. The experimental setup for the capillary experiment using a 35 mm glass bottom petriplate for the observation of nanotube population at box marked position.

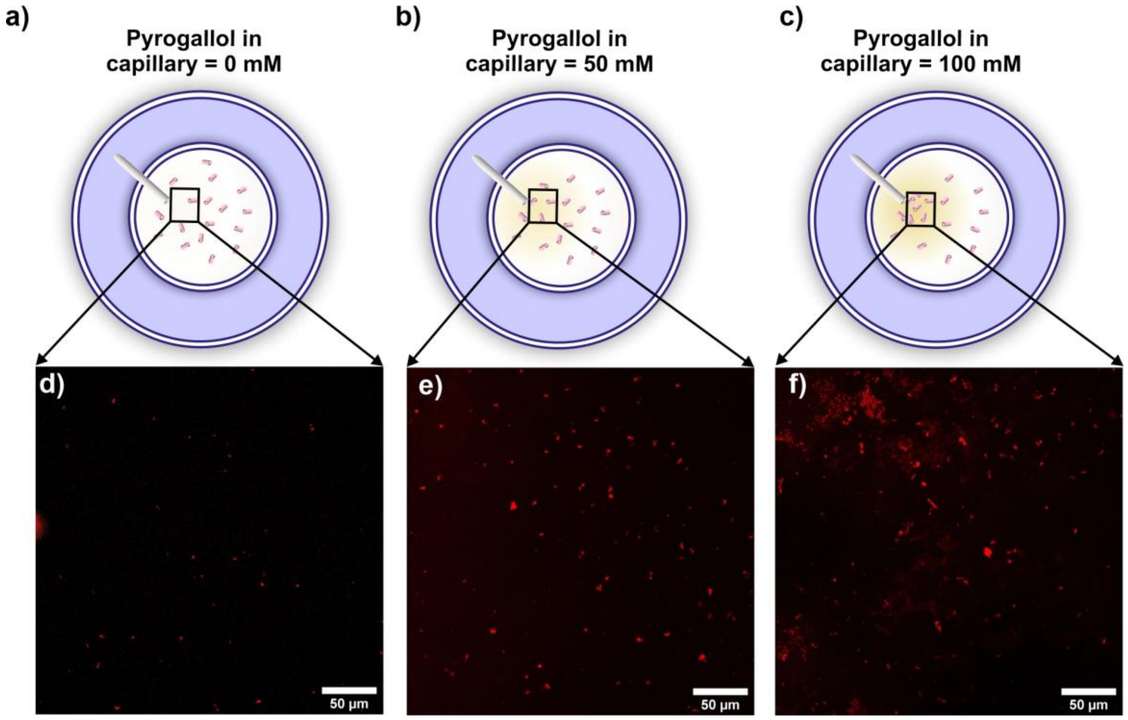

Supplementary Figure 25. (a-c) Experimental setup and confocal micrographs near the opening of the capillaries loaded with (d) 0 mM, (e) 50 mM, and (f) 100 mM pyrogallol after 15 min.

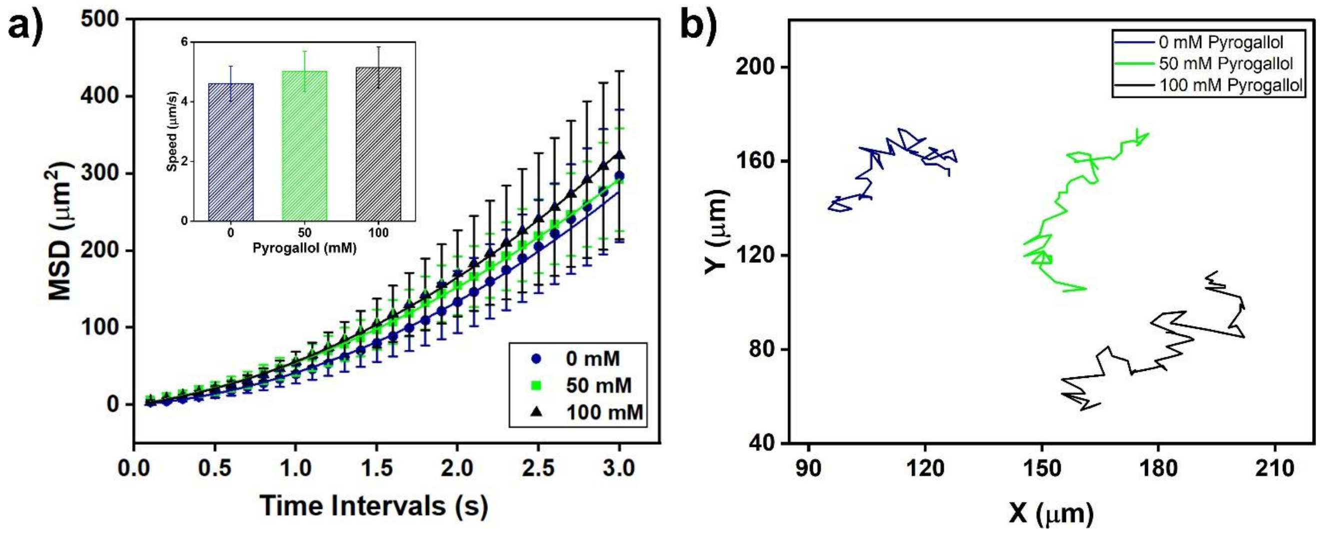

Supplementary Figure 26. (a) MSD vs time interval with varying pyrogallol concentration (inset shows velocity profile) and (b) tracking trajectories of the chemotactic motility of dual enzyme loaded nanotubes.

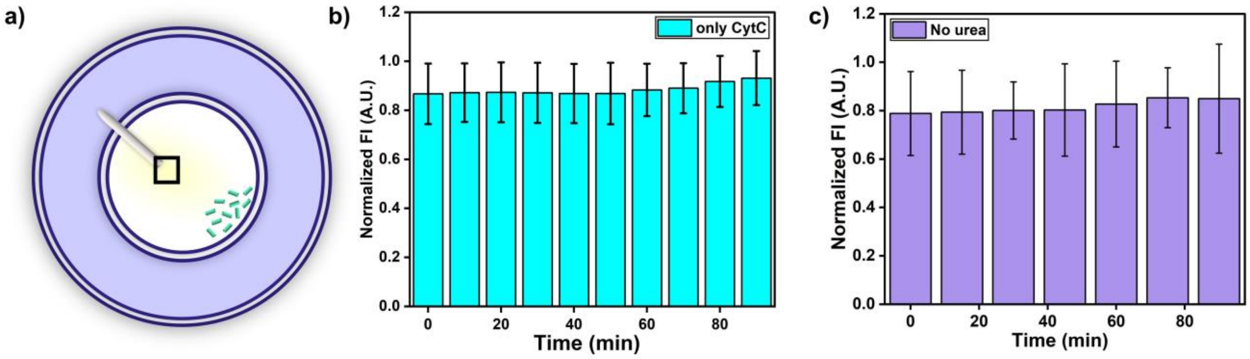

Supplementary Figure 27. (a) Experimental setup. Bar diagrams showing change in normalized fluorescent intensity of (b) only CytC-loaded nanotubes (in presence of only H_2O_2 and pyrogallol gradient) and (c) urease-CytC loaded nanotubes in absence of urea (in presence of only H_2O_2 and pyrogallol gradient), near the opening of the capillaries.

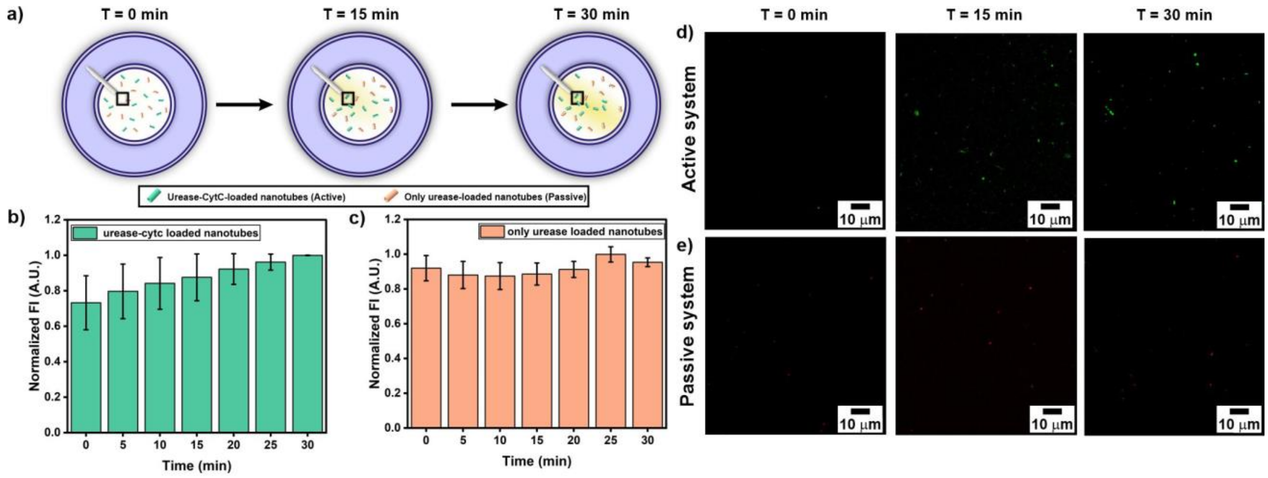

Supplementary Figure 28. (a) Schematic representation of the chemotaxis observed in the mixed system of active and passive nanotubes. Change in normalized fluorescent intensity of (b) active (urease-CytC loaded) and (c) passive nanotubes (only urease loaded). Corresponding CLSM images of the (d) active and (e) passive systems.

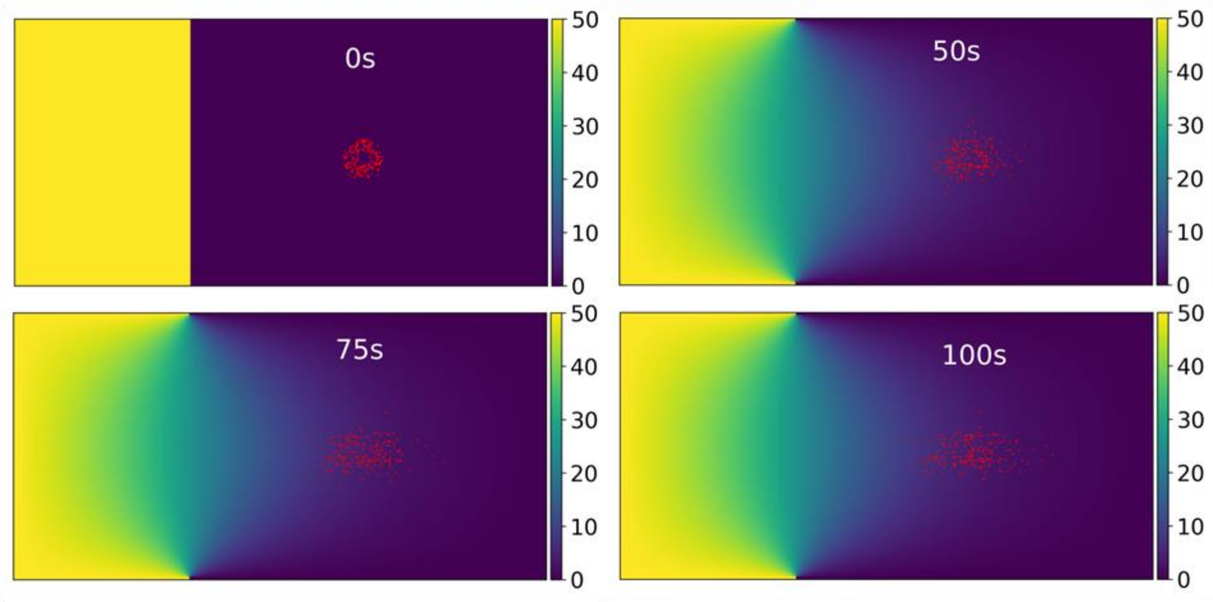

Supplementary Figure 29. Snapshots show spatial profiles of nanotubes in absence of urea. Nanotubes are represented in color red and the color bar represents the concentration of pyrogallol

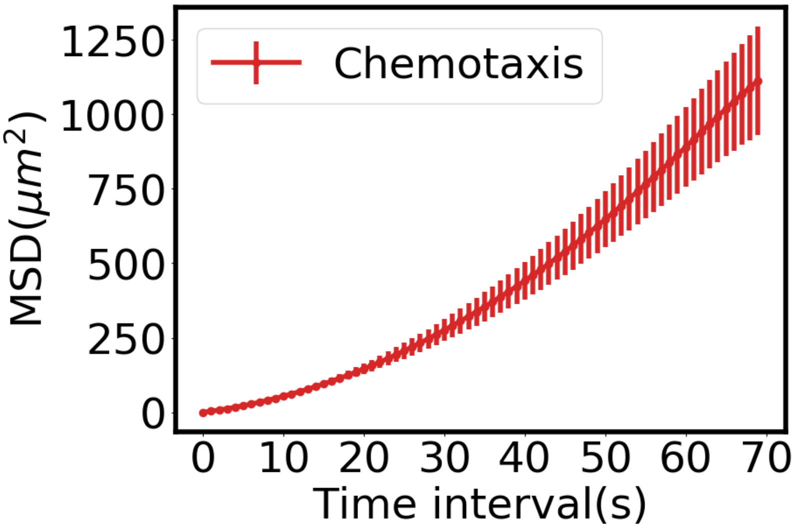

Supplementary Figure 30. MSD as a function of time interval in presence of dual enzyme (urease + CytC) and the substrates from simulated data.

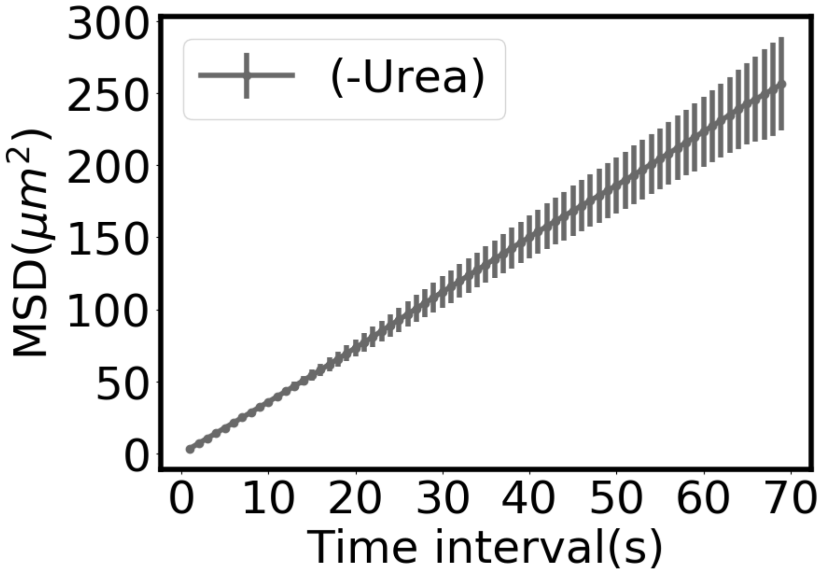

Supplementary Figure 31. MSD as a function of time interval for dual enzyme loaded amylobots in absence of urea (control simulation)

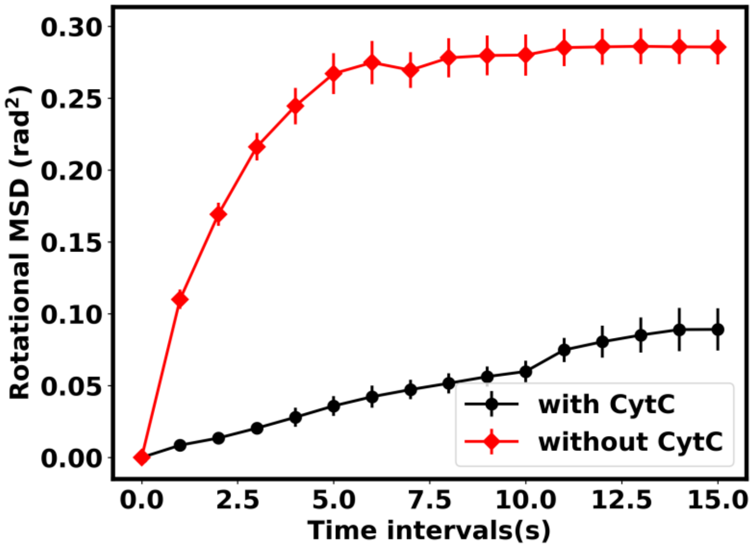

Supplementary Figure 32. Computationally derived rotational MSD of the nanotubes as a function of time interval, in the presence and absence of CytC (control simulation).

Supplementary References

1. Somasundar, A., Ghosh, S., Mohajerani, F., Massenburg, L. N., Yang, T., Cremer, P. S., Velegol, D. & Sen, A. Positive and negative chemotaxis of enzyme-coated liposome motors. *Nat. Nanotech.* **14**, 1129-1134 (2019).
2. Zhang, Y. & Hess, H. Chemically-powered swimming and diffusion in the microscopic world. *Nat. Rev. Chem.* **5,** 500–510 (2021).
3. Song, S., Llopis-Lorente, A., Mason, A. F., Abdelmohsen, K. E. A. L. & van Hest, J. C. M. Confined motion: motility of active microparticles in cell-sized lipid vesicles. *J. Am. Chem. Soc.* **144**, 30, 13831–13838 (2022).
4. Bolleter, W. T., Bushman, C. J. & Tidwell, P. W. Spectrophotometric determination of ammonia as indophenol. *Anal. Chem.* **33**, 592–594 (1961).
5. Patton, C. J. & Crouch, S. R. Spectrophotometric and kinetics investigation of the Berthelot reaction for the determination of ammonia. *Anal. Chem.* **49**, 464–469 (1977).
6. Kapil, N., Singh, A. & Das*,* D. Cross-β Amyloid nanohybrids loaded with cytochrome c exhibit superactivity in organic solvents. *Angew. Chem*. *Int. Ed*. **54**, 6492-6495 (2015).
7. Kapil, N., Singh, A., Singh, M. & Das, D. Efficient MoS2 exfoliation by cross-β-amyloid nanotubes for multistimuli-responsive and biodegradable aqueous dispersions. *Angew. Chem*. *Int. Ed.* **55**, 7772-7776 (2016).
8. Wang, Q., Yang, Z., Zhang, X., Xiao, X., Chang, C. K. & Xu, B. A supramolecular-hydrogel-encapsulated hemin as an artificial enzyme to mimic peroxidase. *Angew. Chem*. *Int. Ed*. **46**, 4285–4289 (2007).
9. a) Rana, N., Ghosh, P. & Perleker, P. Spreading of nonmotile bacteria on a hard agar plate: Comparison between agent-based and stochastic simulations. *Phys. Rev. E*. **96**, 052403 (2017). b) Bera, P., Wasim, A., Mondal, J. & Ghosh, P. Mechanistic underpinning of cell aspect ratio-dependent emergent collective motions in swarming bacteria. *Soft Matter* **17**, 7322-7331 (2021).
10. Usov, I. & Mezzenga, R. Correlation between nanomechanics and polymorphic conformations in amyloid fibrils. *ACS Nano* **8**, 11035-11041 (2014).
11. Yu, L., Banerjee, I. A., Gao,X., Nuraje, N. & Matsui, H. Fabrication and application of enzyme-incorporated peptide nanotubes. *Bioconjugate Chem.* **16**, 1484−1487 (2005).
