# Supplementary material for: Dual enzyme-powered chemotactic cross β amyloid based functional nanomotors
Source: Nat Commun. 2023 Sep 22;14:5903. doi: 10.1038/s41467-023-41301-x (PMC10516904; doi:10.1038/s41467-023-41301-x)
Supplement: Supplementary file 3 — Description of Additional Supplementary Files [file 41467_2023_41301_MOESM3_ESM.pdf]

Supplementary Movie descriptions

Supplementary Movie 1. Motion of amyloid based nanomotors in absence of urea.

Supplementary Movie 2. Motion of amyloid based nanomotors in 25 mM of urea.

Supplementary Movie 3. Motion of amyloid based nanomotors in 50 mM of urea showing the trajectory of 5 nanomotors.

Supplementary Movie 4. Motion of amyloid based nanomotors in 100 mM of urea showing the trajectory of 5 nanomotors.

Supplementary Movie 5. Motion of amyloid based nanomotors not loaded with urease in presence 100 mM of urea (control study).

Supplementary Movie 6. Motion of urease bound non-sonicated Ac-KL nanotubes in presence of 50 mM urea (control study).

Supplementary Movie 7. Motion of amylobots in absence of urea (Simulated).

Supplementary Movie 8. Motion of amylobots in presence of motility force $\boldsymbol{f_u} = \mathbf{6} \times \mathbf{10^5}\ \boldsymbol{Pa.\mu m^2}$.(Simulated).

Supplementary Movie 9. Motion of amylobots in presence of motility force $\boldsymbol{f_u} = \mathbf{12} \times \mathbf{10^5}\ \boldsymbol{Pa.\mu m^2}$.(Simulated).

Supplementary Movie 10. Motion of amylobots in presence of motility force $\boldsymbol{f_u} = \mathbf{24} \times \mathbf{10^5}\ \boldsymbol{Pa.\mu m^2}$. (Simulated).

Supplementary Movie 11. Motion of CytC bound short Ac-KL nanotubes in presence of substrate pyrogallol (control study).

Supplementary Movie 12. Motion of dual enzyme (urease + CytC) loaded amylobots in presence of urea. (Simulated).

Supplementary Movie 13. Motion of dual enzyme loaded amylobots in the absence of urea (Simulated).
